# The StkSR Two-Component System Influences Colistin Resistance in *Acinetobacter baumannii*

**DOI:** 10.3390/microorganisms10050985

**Published:** 2022-05-08

**Authors:** Sarah K. Giles, Uwe H. Stroeher, Bhavya Papudeshi, Robert A. Edwards, Jessica AP. Carlson-Jones, Michael Roach, Melissa H. Brown

**Affiliations:** 1 College of Science and Engineering, Flinders University, Adelaide, SA 5042, Australia; uwe.stroeher@flinders.edu.au (U.H.S.); nala0006@flinders.edu.au (B.P.); robert.edwards@flinders.edu.au (R.A.E.); jessica.carlsonjones@flinders.edu.au (J.A.C.-J.); michael.roach@flinders.edu.au (M.R.); 2 Research and Development, Neutrog Australia Pty Ltd., Kanmantoo, SA 5252, Australia

**Keywords:** *pmrCAB*, hydrophobicity, lipid A, adherence, phosphoethanolamine, TCS, antibiotic resistance

## Abstract

*Acinetobacter baumannii* is an opportunistic human pathogen responsible for numerous severe nosocomial infections. Genome analysis on the *A. baumannii* clinical isolate 04117201 revealed the presence of 13 two-component signal transduction systems (TCS). Of these, we examined the putative TCS named here as StkSR. The *stkR* response regulator was deleted via homologous recombination and its progeny, Δ*stkR*, was phenotypically characterized. Antibiogram analyses of Δ*stkR* cells revealed a two-fold increase in resistance to the clinically relevant polymyxins, colistin and polymyxin B, compared to wildtype. PAGE-separation of silver stained purified lipooligosaccharide isolated from Δ*stkR* and wildtype cells ruled out the complete loss of lipooligosaccharide as the mechanism of colistin resistance identified for Δ*stkR*. Hydrophobicity analysis identified a phenotypical change of the bacterial cells when exposed to colistin. Transcriptional profiling revealed a significant up-regulation of the *pmrCAB* operon in Δ*stkR* compared to the parent, associating these two TCS and colistin resistance. These results reveal that there are multiple levels of regulation affecting colistin resistance; the suggested ‘cross-talk’ between the StkSR and PmrAB two-component systems highlights the complexity of these systems.

## 1. Introduction

*Acinetobacter baumannii* is a Gram-negative nosocomial opportunistic pathogen that causes severe infections worldwide and is at the top of the World Health Organization’s (WHO) list of priority pathogens for the R&D of new antibiotics [1]. Pandemic outbreaks of *A. baumannii* are increasing in incidence and severity due to its multidrug and pandrug-resistant nature [2,3,4,5]. Patients presenting with these infections commonly rely on “last resort” antibiotics such as the polymyxin class of drugs. Polymyxin E (colistin) and polymyxin B are cationic peptides composed of a cyclic heptapeptide covalently attached to a fatty acyl chain [6,7]. Colistin is bactericidal with positively charged ɑ,ɣ-diamonobutyric acid residues which interact by attaching and displacing the magnesium and calcium divalent cations of the negatively charged phosphate groups of lipid A. Lipid A stabilizes the lipooligosaccharide or lipopolysaccharide component of the bacterial membrane [8,9,10]. Displacement of these cations increases the permeability of the outer membrane causing uptake into the periplasm and perturbation and surface corrugation of the outer and inner membranes. This can result in the collapse of the outer membrane and leakage of the cellular content, a phenomenon known as blebbing [11,12].

For *A. baumannii* and other pathogenic bacteria, the ability to sense and respond to external and internal signals is critical for survival in the environment as well as within the host. Two-component signal transduction systems (TCS) aid in the regulation of pathogenesis and thus are implicated in the circumvention of antibiotic treatments [5,8,13,14,15]. Regulation of these systems typically involves a membrane-bound histidine kinase and its cognate response regulator, however, ‘cross-talk’ between histidine kinases and non-cognate response regulators also occurs [16,17]. When a signal is received by a histidine kinase, the histidine kinase protein forms a dimer and is autophosphorylated at a conserved histidine residue. The phosphate molecule activates its cognate response regulator protein by being transferred to the conserved aspartate residue in the N-terminal region [5,18,19]. Activation of the response regulator typically produces a conformational change in the C-terminal or variable region of the response regulator protein [18], and in the case of DNA-binding response regulators, this allows for stimulation or repression of target genes thereby affecting cellular processes [19,20].

TCS are associated with alterations in antibiogram profiles; for example, in *A. baumannii* the PmrAB system is linked to increased polymyxin resistance, where mutations in *pmrA* and/or *pmrB* result in up-regulation of the *pmrCAB* operon leading to the modification of lipid A by the addition of phosphoethanolamine (pEtN) to either 4′ or 1′ phosphate lipid A [5,21,22,23]. Other two-component systems implicated in antibiotic resistance include the AdeRS system which regulates an efflux pump which significantly contributes to antibiotic resistance of aminoglycosides and tigecycline in *A. baumannii* [5,24]. Additionally, in other bacteria like *Klebsiella pneumoniae*, TCS cascades have been identified in the PhoPQ and the PmrAB systems involved in regulating colistin resistance via the *pmrCAB* operon [25,26]. 

Two main mechanisms for colistin resistance have been previously identified in *A. baumannii* strains. One is the complete loss of the lipid A portion of the lipooligosaccharide, which correlates with mutations in *lpxA*, *lpxC*, *lpxD*, *lpsB*, *lpsD*, and *vacJ* genes which encode acyltransferases essential to lipid A biosynthesis associated with permeability defects [8,9,27,28]. The second is the modification of lipooligosaccharide by the addition of pEtN or 4-amino-4-deoxy-l-arabinose to the phosphate groups of lipid A [2,8]. Two distinct pathways for this modification have been identified; the first requires *pmrC* encoded in an operon with the PmrAB two-component system [10,27,28,29,30,31]. The second is correlated to the insertion of an IS element (IS*Aba125*) in an H-NS-family transcriptional regulator, increasing expression of *eptA* which encodes a second pEtN transferase [32]. The increase in pEtN reduces the overall membrane electronegativity, thereby reducing its affinity to polymyxins [23]. Here we identified a novel regulator that influences the expression of *pmrCAB* elucidating that multiple layers of regulation can be employed by *A. baumannii* to confer colistin resistance.

## 2. Materials and Methods

### 2.1. Bacterial Strains and Media

The clinical *A. baumannii* bacterial strains used in this study have been described previously [33] and were cultured as previously described [34]. For maintenance, conjugation, cloning, and replicating plasmids, *Escherichia coli* DH5α, SM10, or JM109 cells were used [35,36,37]. The *A. baumannii* 04117201 isolate belongs to the International Clone type II lineage and was obtained from a tracheal aspirate sample at Flinders Private Hospital, SA, Australia.

### 2.2. Eukaryotic Cell Adherence Assay

Adherence of *A. baumannii* to human type 2 A549 pneumocytes was investigated as previously described [34]. The data collected for the adherence assays were obtained from at least three independent experiments and represent data points from each experiment in quadruplicate wells.

### 2.3. Minimum Inhibitory Concentration and Disk Diffusion Susceptibility

Minimum inhibitory concentrations (MIC) were determined using standard methods as previously described [38]. In brief, 96-well microtiter trays containing a 2-fold dilution series of the compound of interest were prepared and cultures added 1:1 in Mueller-Hinton broth (MH) (Oxoid, ThermoFisher, Adelaide, SA, Australia) before incubating overnight at 37 °C with shaking (~50 rpm). Bacterial growth was examined by absorbance at OD_600_ using a Multiskan EX (Adelab Scientific, Adelaide, SA, Australia). The standard agar disc diffusion technique was performed on MH 1% agar with bacterial strains diluted to the McFarland 0.5 standard. Results of the Kirby–Bauer disc diffusion were evaluated according to the Clinical and Laboratory Standards Institute guidelines [39,40].

### 2.4. Deletion Replacement Mutant Construction by Homologous Recombination

Construction of *A. baumannii* 04117201 *stkR*-inactivated derivatives was undertaken by homologous recombination as described previously [24] with the following modification. DNA fragments corresponding to 1.2 kb upstream and 1 kb downstream of *stkR* were PCR amplified using VELOCITY^TM^ DNA polymerase (Bioline, Sydney, NSW, Australia) using specific oligonucleotides designed in PRIMER Biosoft NetPrimer (www.premiersoft.com, accessed on 22 September 2015) (Table S1). Amplified PCR products of the appropriate size were cloned into the suicide vector pEX18Tc via *Xba*I, *Bam*HI, and *Sac*I restriction sites and transformed into *E. coli* DH5α cells. Following sequence confirmation, pEX18Tc clones were electroporated into freshly prepared electrocompetent *A. baumannii* 04117201 bacterial cells and homologuous recombination was undertaken as previously described [41]. Two Δ*stkR* derivatives were generated, and the phenotypic analysis of one is presented here. 

### 2.5. Cell Surface Hydrophobicity Tests

Cell surface hydrophobicity was examined as described previously [34,41]. The OD_600_ of the cell suspension was determined before (OD_initial_) and after (OD_final_) the addition of xylene. The hydrophobicity index was calculated as (OD_initial_ − OD_final_/OD_initial_) × 100. Quantitative data were collected from at least three experiments from three different days.

### 2.6. Transmission Electron Microscopy

Transmission electron microscopy (TEM) was undertaken as previously described [42] with the following modifications. *A. baumannii* cells grown to log phase were treated for 1 h at 37 °C with shaking in the presence of colistin at 0, 3, or 12 µg/mL. Washed and fixed samples were dehydrated in an ethanol series and embedded in 1 mL EMbed 812/Araldite 502 resin (Emgrid, Adelaide, SA, Australia). Samples were cut with a diamond knife (Diatome, Philadelphia, PA, USA) and microtome (Leica EM UC6 Ultramicrotome) (Leica, Sydney, NSW, Australia) and examined using a TEM (FEI Tecnai G2 Spirit, New York, NY, USA). A magnification of 60,000× was used for bacterial cell analysis, whereas for determination of blebs per cell 30 TEM slides (~600 cells) at a magnification of 11,500× was used. A total of 30 independent fields, including a minimum of 10 cells per field of view, spanning three different slides, were used to calculate blebs/cell. 

### 2.7. SDS-PAGE and Lipooligosaccharide Silver Staining

SDS-PAGE and lipooligosaccharide silver staining was undertaken as previously described [43,44]. In short, Proteinase K treated samples were resolved on an SDS-PAGE gel. Fixation was achieved using ethanol and glacial acetic acid and followed by oxidization using ethanol and glacial acetic acid and periodic acid. The gel was washed with MilliQ and stained with NaOH, NH_4_OH and AgNO_3_. 

### 2.8. RNA Isolation

RNA was extracted as previously described [34] with the following modifications. Cells were grown in MH broth (Oxoid, ThermoFisher, Adelaide, SA, Australia) to an OD_600_ of 0.6 and treated with colistin at 3 μg/mL for 1 h. Harvested bacterial cells were lysed and TRizol and chloroform treated. Phases were separated by centrifugation and RNA extracted using the RNA Mini Kit II (Bioline, Australia) following the manufacturer’s recommendations before DNaseI treatment.

### 2.9. Quantitative Reverse Transcription PCR

The iScript reverse-transcriptase cDNA synthesis kit (Bio-Rad, Sydney, NSW, Australia) was used to synthesize cDNA following the manufacturer’s recommendations. Quantitative reverse transcription PCR was performed using iTaq^TM^ Universal SYBR^®^ green supermix (Bio-Rad, Sydney, NSW, Australia) in conjunction with a Rotor-Gene Q (Qiagen, Melbourne, VIC, Australia) with oligonucleotides designed in PRIMER Biosoft NetPrimer (www.premiersoft.com, accessed on 10 July 2016) (Table S1). A typical quantitative reverse transcription PCR run was 2 min at 95 °C followed by 40 cycles of 10 s at 95 °C, 15 s at 60 °C, and 20 s at 72 °C [45]. Transcriptional differences were calculated using the 2^−ΔΔ*CT*^ method [46] and the 16S rRNA (A1S_r01) and *GAPDH* (A1S_2501) transcription levels were used as a reference.

### 2.10. DNA Extraction

Bacterial cultures of *A. baumannii* 04117201 and Δ*stkR* were grown in MH broth to an OD_600_ of 0.6. DNA purification was conducted using a spin column purification kit (NucleoSpin tissue, Macherey-Nagel, Düren, Germany) following manufacturer’s instructions. Library preparation for sequencing on a MinION using a Flongle Flow cell and adaptor (Oxford Nanopour, Düren, UK) was undertaken following the SQK-RBK004 protocol as per the manufacturer’s instructions.

### 2.11. Library Quality Control and Bioinformatics

Base calling was achieved using guppy-basecaller converting fast5 files to fastq format, along with demultiplexing and adapter trimming. Filtlong v0.2.0 (https://github.com/rrwick/Filtlong, accessed on 15 January 2021) was used to remove reads less than 1000 bp and 5% of the lowest quality reads. Post quality control (QC), Nanopore reads were de novo assembled using Unicycler v0.4.8 (https://github.com/rrwick/Unicycler, accessed on 20 January 2021) [47] producing a complete chromosome for the 04117201 wild-type (WT) strain and the Δ*stkR* mutant strain. To correct the indels in the assembly, Pilon v1.23 [48] was run to polish the complete assemblies with RNAseq Illumina reads. To confirm the quality of the assembly improved, the pre pilon assembly and post pilon assembly was run through ideel (https://github.com/mw55309/ideel, accessed on 27 January 2021). Ideel calculates the ratio of the protein sequences predicted in the assemblies compared to the top protein hits they aligned against in the Uniprot database. Finally, Bandage was used to visualize the completed assembly [49]. To identify the variants between strains, reads from the 04117201 WT strain and two Δ*stkR* mutant strains (Section 2.4) were aligned against the complete 04117201 WT strain assembly using Bowtie2 v2.3.5 [50]. The mapped reads were piled up against the reference 04117201 WT strain using SAMtools v1.9 [51]. VCFools v0.1.17 [52] was used to identify the SNPs observed in the alignment. To identify which genes the SNPs were found in, the assembled genomes were annotated using Prokka v1.14.6 [53] and validated using progressive Mauve (v2) and Artemis.

### 2.12. Statistical Analysis

The Graphpad Prism 7.0 (La Jolla, CA, USA) statistical package was used to analyze the results where appropriate. Assessments of skewness, kurtosis, and the Shapiro–Wilk normality test [54] were undertaken on all measures, and non-parametric tests were applied as needed.

### 2.13. Accession Numbers

Sanger sequencing of the DNA sequence of the two-component signal transduction system StkRS of *A. baumannii* 04117201 has been deposited in Genbank ID 2135660.

## 3. Results

### 3.1. Selection of A. baumannii Isolate

**Figure 1:**
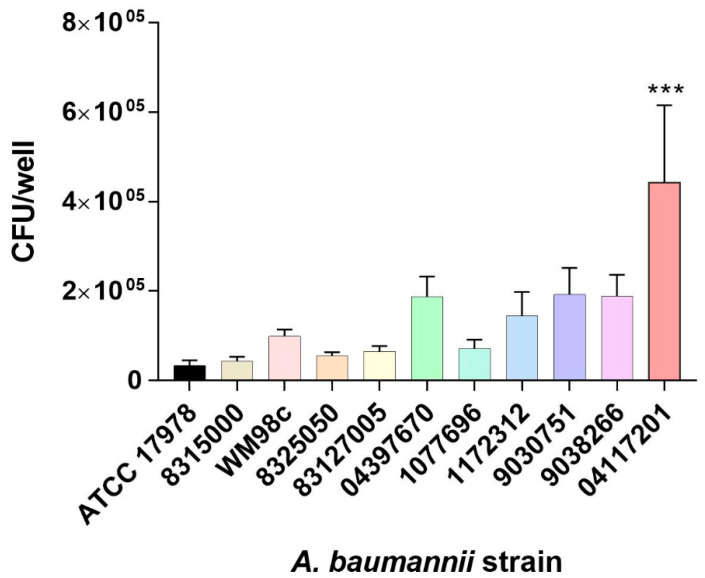
Cell adherence capacity of *A. baumannii* clinical strains to A549 pneumocytes. The CFU/well values of 11 *A. baumannii* strains were examined after exposure to A549 cells. Bars indicate the standard error of three separate experiments. Assessments of skewness, kurtosis and the Shapiro–Wilk normality test were undertaken. *** represents a *p*-value of < 0.001 as determined by an analysis of variance test, between ATCC 17978 and the clinical isolate.

The commonly used reference strain *A. baumannii* ATCC 17978 is not highly virulent in our hands, as seen in Figure 1 and Table 1, therefore we sought to identify a potentially virulent *A. baumannii* isolate. Within our collection, 11 clinical isolates were screened for two important clinical virulence-related phenotypes: cell adherence and antibiotic resistance [55]. Incubation with human A549 pneumocytes revealed the *A. baumannii* isolate 04117201 to be significantly more adherent than the reference ATCC 17978 strain (*p* < 0.001; Figure 1), and more adherent than the other clinical strains tested (Figure 1). 

Antimicrobial resistance screening of the 11 clinical isolates identified the 04117201 strain as more resistant to many of the compounds tested compared to the reference ATCC 17978 strain. However, it is sensitive to erythromycin (Table 1), allowing the use of this antibiotic as a selection marker for mutant construction. As previously published, we have shown that the *A. baumannii* 04117201 cells can form a well-established biofilm, which is an important virulence factor [56]. 

### 3.2. Construction of an A. baumannii 04117201ΔstkR Derivative

The genome of the WT *A. baumannii* 04117201 strain was sequenced by Sanger sequencing at the Welcome Trust Sanger Institute and assembled using Velvet v1.2.03. Visual inspection of the sequence using RAST v2.0 identified 13 TCS response regulator proteins in the genome. A comparison of the 13 TCS identified in the clinical 04117201 strain was undertaken against those present in the *A. baumannii* SDF and ATCC 17978 strains identifying five response regulators proteins AdeR, BarA, StkR and two orphan response regulators NasT and RsbU not present in the SDF avirulent strain (Table 2). The TCS not present in *A. baumannii* SDF we named as StkSR ‘sticky’ due to the observed increased adherence to eukaryotic cells. The StkSR system identified in the clinical 04117201 strain encompasses a membrane bound hybrid histidine kinase and a cytosolic response regulator. The StkR response regulator has a conserved C-terminal helix-turn-helix motif placing it in the LuxR family. To assess the role of the StkSR system in the clinical 04117201 strain, two independent *A. baumannii* Δ*stkR* mutants were constructed. One Δ*stkR* mutant was selected and its growth kinetics compared to the WT in different media representing different types of environment conditions including, MH (laboratory), minimal M9 medium (desiccation on surfaces and ventilator tubing), lung medium (pneumonia) [57], and whole sheep blood (septicemia) (Figure S1). Growth of Δ*stkR* was comparable to WT in MH, M9 and lung medium. However, bacterial growth was significantly reduced in whole sheep’s blood. Both the WT and Δ*stkR* strains showed a 5-log decrease in colony forming units (CFU) in the first hour post exposure to whole sheep’s blood, after which the CFU of both strains did not change for 2 h. The Δ*stkR* mutant strain showed limited recovery and reached 10^4^ CFU by the 4-h time point, whereas the WT did not reach a 10^4^ CFU for another hour; both the WT and Δ*stkR* strains recovered to 10^6^ CFU by 7 h. These data indicate that the deletion of the response regulator *stkR* does not result in a general decrease in bacterial cell fitness when grown in MH, M9 or lung media; however, there is a marked increase in bacteria cell CFU recovery of the Δ*stkR* mutant strain compared to the WT strain after exposure to whole sheep’s blood.

### 3.3. Assessment of the A. baumannii ΔstkR Mutant Strain Antibiogram

As TCS are known for regulating a variety of genes involved in antibiotic resistance, the Δ*stkR* and WT strains were assessed for their ability to resist a variety of antimicrobial compounds by MIC assays (Table 3). Five of the 16 compounds tested had altered antibiograms between the WT and Δ*stkR* strains. There was at least a two-fold increase in resistance to colistin and polymyxin B (both cationic antimicrobial compounds) for Δ*stkR*. Chloramphenicol and rifampicin showed a two-fold increase of the Δ*stkR* mutant compared to the WT strain and kanamycin resistance increased past the MIC threshold of detection in the Δ*stkR* mutant compared to the WT strain (Table 3).

### 3.4. Examination of Cell Surface Hydrophobicity of the A. baumannii ΔstkR Mutant Strain When Exposed to Sub-MIC Levels of Colistin

**Figure 2:**
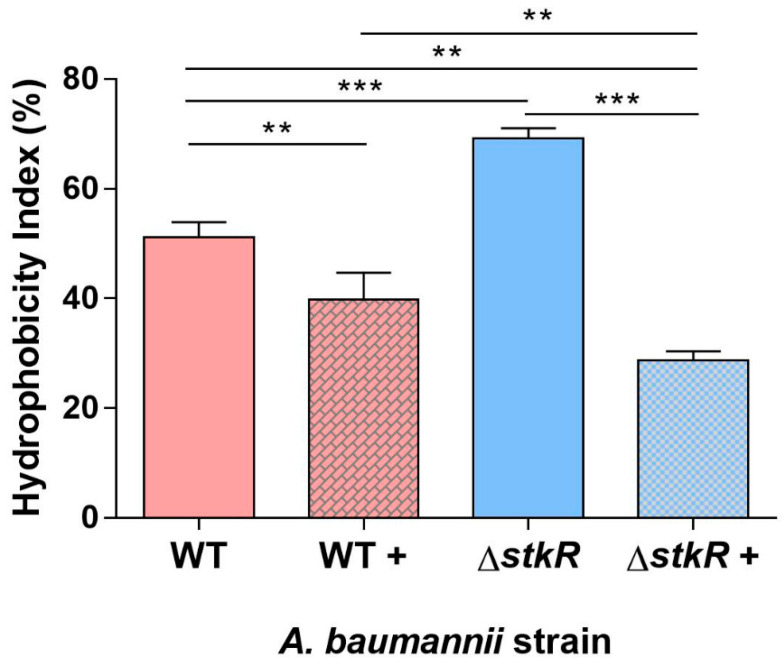
Cell surface hydrophobicity of *A. baumannii* 04117201 and Δ*stkR* mutant. Cell surface hydrophobicity was assessed by the cells’ affiliation to xylene. *A. baumannii* strain 04117201 (WT) is without colistin exposure, WT + is with exposure to 3 μg/mL of colistin. *A. baumannii* 04117201 Δ*stkR* mutant is without colistin exposure, Δ*stkR*+ is with exposure to 3 μg/mL of colistin. Bars represent the standard deviation of three separate experiments. A Kruskal–Wallis test was performed to compare the means of the WT and Δ*stkR* mutant with and without colistin exposure. *** represents a *p*-value < 0.001 and ** represents a *p*-value of <0.01 as established by an analysis of variance test.

Colistin is a cationic antimicrobial compound that acts by attaching and displacing the magnesium and calcium cations and thereby destabilizing the lipooligosaccharide in *A. baumannii* [69]. The hydrophobicity of the cell surface influences the affinity of colistin to the membrane [8,70]. Therefore, the cell surface hydrophobicity of the Δ*stkR* mutant strain was compared with the WT by its affinity to xylene with and without exposure to sub-MIC levels of colistin [41,71]. Under no colistin stress the Δ*stkR* mutant strain showed a higher cell surface hydrophobicity compared with the WT strain (*p* < 0.001; Figure 2), however, under sub-MIC levels of colistin, the Δ*stkR* mutant strain showed a significant decrease in cell surface hydrophobicity compared with the WT strain (*p* < 0.01; Figure 2). These results demonstrate the ability of the StkR response regulator to alter the bacterial cells surface hydrophobicity in response to external stressors.

### 3.5. Visualizing the Effects of Colistin Stress on the Bacterial Cell Envelope

**Figure 3:**
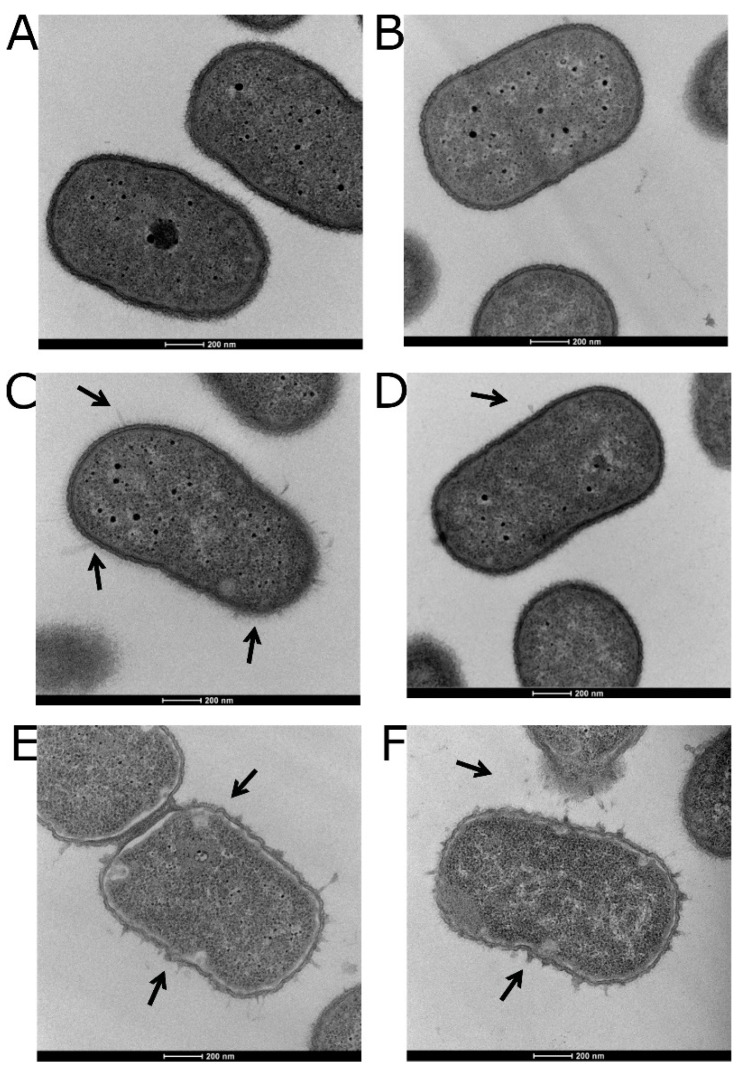
Membrane perturbation of *A. baumannii* 04117201 (WT) and Δ*stkR* mutant after colistin stress. WT and Δ*sktR* mutant strains were subjected to sub-MIC and MIC levels of colistin (3 μg/mL and 12 μg/mL) and cross-sections were examined by TEM (60,000× magnification); 200 nm scale is shown in white. Images are of (**A**) WT, (**B**) Δ*sktR*, (**C**) WT after exposure to 3 μg/mL colistin, (**D**) Δ*stkR* after exposure to 3 μg/mL of colistin, (**E**) WT after exposure to 12 μg/mL colistin, and (**F**) Δ*stkR* after exposure to 12 μg/mL colistin; arrows show membrane perturbation and/or leakage of cellular contents.

As the mode of action of colistin involves the disruption of the cell surface lipopolysaccharide [72], changes to the bacterial cell surface were assessed by TEM. Neither the WT nor Δ*stkR* mutant strain showed disruption of the bacterial cell outer membrane without colistin treatment (Figure 3A,B). However, after exposure to a sub-MIC level of colistin (3 µg/mL) (Figure 3C,D) significantly more protruding events per bacterial cell (blebs/cell) were observed in the WT strain (2.5 ± 0.3 blebs/cell) compared to the Δ*stkR* strain (0.7 ± 0.05 blebs/cell; *p* < 0.0001) [23,73]. Treatment of the WT and Δ*stkR* mutant strains with 12 µg/mL of colistin resulted in both strains exhibiting extensive membrane perturbation leading to a breakdown of the bacterial cell wall with partially disintegrated cells (Figure 3E,F).

### 3.6. Visualizing the Lipooligosaccharide Composition of the A. baumannii ΔstkR Mutant and WT Strain

**Figure 4:**
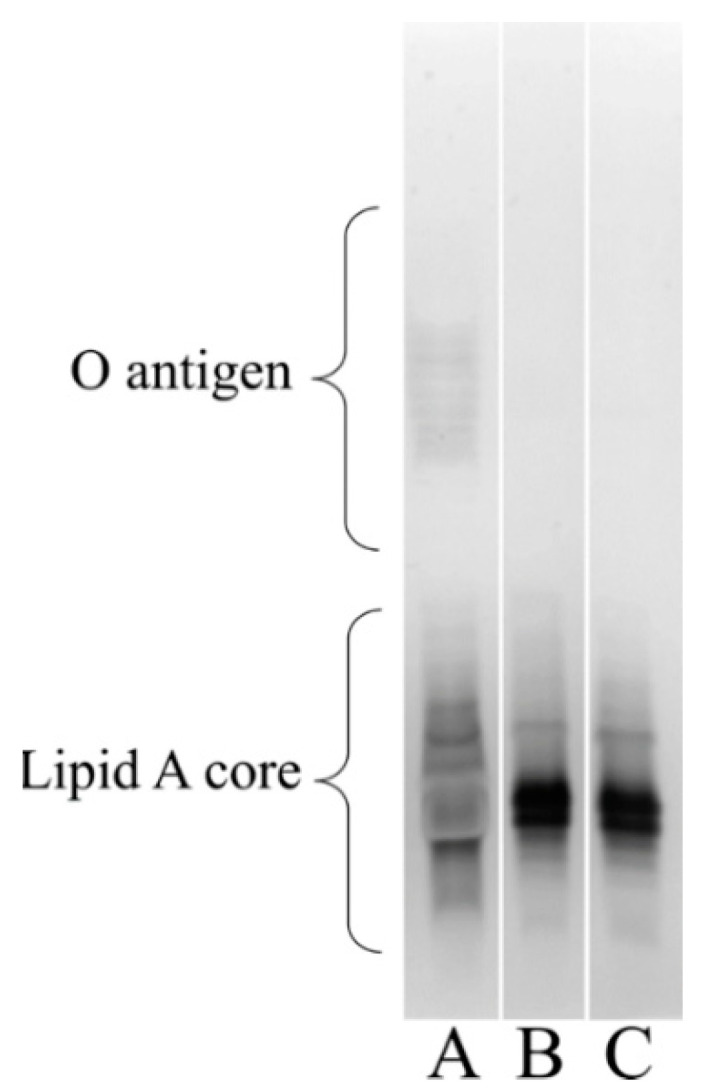
Lipooligosaccharide analysis of the 04117201 (WT) and Δ*stkR* mutant. The lipid A and O-antigen components of the strains’ membrane was resolved on a 15% polyacrylamide gel and silver stained. (**A**) control lipopolysaccharide from *Pseudomonas aeruginosa* PA01 showing the presence of O-antigen; (**B**) WT, and (**C**) Δ*stkR*. Brackets identify either the lipid A core or O-antigen regions.

*A. baumannii* acquires resistance to colistin by two known mechanisms: one is the complete loss of the lipid A portion of lipooligosaccharide [70,74] and the other is through the modification of lipid A [75]. To see if disruption of *stkR* influences lipid A production, silver staining of the bacterial cell lipooligosaccharide was performed. The WT and Δ*stkR* bacterial strains were grown under normal conditions and their lipid A profiles assessed. Colistin resistance cannot be attributed to the loss of lipid A as both the WT and Δ*stkR* cells were visually observed to be identical (Figure 4).

### 3.7. Transcriptional Profiling of the A. baumannii ΔstkR Mutant and WT Strain Treated with Sub-MIC Concentrations of Colistin

The PmrAB TCS regulates *pmrC,* whose product is responsible for lipid A modification by the addition of pEtN leading to an increase in colistin resistance [74,75]. Quantitative reverse transcription polymerase chain reaction (qRT-PCR) of the *pmrA*, *pmrB* and *pmrC* genes identified an increase in transcription of these genes in the Δ*stkR* mutant strain when exposed to the sub-MIC of colistin, by 6.63-fold (*p* < 0.02), 8.22-fold (*p* < 0.0001) and 17.63-fold (*p* < 0.0003), respectively, compared to the WT strain. Increased transcription of *pmrCAB* can be related to point mutations in PmrAB itself [72]. A sequencing analysis was therefore undertaken on the Δ*stkR* mutant strain to identify if any mutations in the *pmrAB* genes were present. Sanger sequencing analysis of the 3.6 kb region encompassing the *pmrCAB* operon identified no point mutants in the Δ*stkR* mutant strain compared to the 

WT (data not shown). This finding suggests that deletion of the *stkR* gene influences the *pmrCAB* operon and is likely a new mechanism influencing colistin resistance.

### 3.8. Comparative Genomes

To determine whether any other mutations may be causing the phenotypes exhibited by the Δ*stkR* mutant strain, the WT *A. baumannii* strain 04117201 was resequenced and the Δ*stkR* mutant strain sequenced using a Nanopore MinION. Two independent Δ*stkR* mutant strains were compared with the WT strain, which identified 14 single nucleotide polymorphisms (SNPs) present in both of the Δ*stkR* strains assessed (Table 4). Out of the 14 SNPs identified, seven are in non-coding regions of the chromosome; two are in repeat regions including a hypothetical protein; two are synonymous and three are non-synonymous. The first of the non-synonymous SNPs is at position 1150782 within a gene annotated as an aldehyde dehydrogenase. This produces an amino acid change from a stop codon to a glutamine, therefore the open reading frame now encodes a fusion of two aldehyde dehydrogenase transcribed together and this mRNA is unlikely to form an active protein. Further analysis of the WT 04117201 identified six copies of aldehyde dehydrogenase in the chromosome. Aldehyde dehydrogenase is implicated in a broad range of metabolic pathways; however, as it is commonly found in multiple copies in the genome, this inactive gene is likely to have minimal, if any, impact on cellular regulation [76]. The second non-synonymous change is at position 3132365 and is annotated as the phenazine biosynthesis protein (PhzF), a secondary metabolite known to be produced in Pseudomonas spp. which correlates to the colour exhibited by the species [77]. Although there is a change from a nonpolar (phenylalanine) to a polar amino acid (serine), this change is close to the C-terminal end of the protein and is unlikely to affect the secondary structure and function. To date, this gene has not been implicated in antibiotic resistance. The final non-synonymous change is at position 3445432, encoding an alcyl-CoA dehydrogenase, a class of enzymes that are associated with oxidation in the mitochondria and not involved in cellular regulation. “The non-coding regions harboring SNPs were also investigated. All were between 26 to 47 bps from the stop or start codon of any potential coding regions, predicting that these mutations would not influence gene transcription. The SNP at position 538323 results in a glutamine to a glycine change in the region annotated as a repeat hypothetical protein. This region is also upstream from an annotated tail protein and downstream of a major capsid protein suggesting that this region is potential a prophage. The other hypothetical protein containing a SNP is at position 3201037 placed downstream of a polymerase sigma factor RpoN and upstream of the outer membrane phospholipid-binding lipoprotein MlaA. No genetic factors have been described that indicate a link between these proteins”.

## 4. Discussion

Two-component systems have gained considerable attention due to their role in modulating a plethora of virulence mechanisms and multidrug resistance. In *A. baumannii* these systems include: BfmRS, which is involved in fitness, capsule production and biofilm formation; AdeRS, which regulates an efflux pump associated with antibiotic resistance; GacSA, which is involved with regulating the phenyl acetic acid catabolic pathway as well as attenuated virulence in a mouse infection model; the BaeSR system, which modulates efflux pumps and has been implicated in cross talk with the AdeRS systems, and the PmrAB system, which is involved in colistin resistance [4,5,26,66,78]. In this study the diverse nature of these systems was explored through the uncharacterized TCS, StkSR. We chose to investigate the role of this system in the clinical isolate 0411201 because of its potential involvement in influencing virulence (Figure 1) (Table 1) and its ability to form strong biofilms [56].

Comparative bioinformatics analysis of the genes encoding potential response regulator proteins in the WT 04117201 *A. baumannii* strain identified five TCS that are absent from the avirulent *A. baumannii* strain SDF and therefore are hypothesized to affect virulence potential (Table 2). These systems included two orphan response regulators, NasT and RsbU, and two previously characterized response regulators, AdeR, which is associated with efflux pump regulation, and BarA, coordinating the control of type IV pili [24,66]. The novel hybrid TCS named here as StkSR was chosen for further phenotypic investigation. 

A knockout mutant was constructed by replacing the *stkR* gene with an erythromycin resistance cassette giving rise to the Δ*stkR* mutant strain. This produced a significant increase in expression of the *pmrC*, *pmrA* and *pmrB* genes in the Δ*stkR* mutant after sub-MIC exposure to colistin, identifying the *stkR* gene as a potential repressor of the genes *pmrA* and *pmrB* either directly or indirectly. Thus, this result has identified a potential regulatory relationship between the StkSR and PmrAB TCS. 

The increased spread of multidrug- and pan-resistant strains of *A. baumannii* is of global concern. As carbapenem resistance increases, clinicians are more reliant on colistin as a ‘last resort’ antibiotic. As a consequence of its use, colistin resistant strains are continuing to be prevalent, which is resulting in an increase in mortality rates [3,74]. Inactivation of the *stkR* response regulator in *A. baumannii* strain 04117201 resulted in a 2-fold increase in resistance to the polymyxins, colistin and polymyxin B (Table 3). Colistin requires unmodified lipooligosaccharide or lipopolysaccharide to be present to exert its antibacterial properties; to date two mechanisms of resistance to colistin have been described in *A. baumannii*, the complete loss of the lipooligosaccharide portion of the outer membrane and modification of phosphate groups of the lipid A by the addition of pEtN or 4-amino-4-deoxy-l-arabinose, both of which reduce the overall membrane electronegativity [2,8,73,79]. Silver staining of purified lipooligosaccharide from the Δ*stkR* mutant and its WT parent identified the presence of lipooligosaccharide and a similar lipid A composition. Therefore, unlike Henry et al. (2012), the increase in colistin resistance could not be attributed to the loss of lipid A or the gross modification of lipid A under normal conditions [80,81]. However, bacterial cells which have a reduced cell surface hydrophobicity also show an increase in colistin resistance [82]. Therefore, cell surface hydrophobicity was assessed in both Δ*stkR* and WT cells. This revealed a significant reduction in the cell surface hydrophobicity of the Δ*stkR* derivative compared to the WT when in the presence of colistin (Figure 2). This suggests that modification of the bacterial cell is decreasing the electronegative of the cell and thereby decreasing the cell surface hydrophobicity. This modification could be a result of the addition of pEtN to lipid A, correlating the altered expression of the *pmrCAB* operon with cell surface hydrophobicity. When there is no colistin stress on the Δ*stkR* and WT bacterial cells there is an increased cell surface hydrophobicity in Δ*stkR* compared to the WT parent, we suggest that modification of the cell surface is only occurring when the WT and Δ*stkR* mutant strains are stressed with sub-MIC concentrations of colistin, showing that the bacterial cell is actively responding to the antibiotic as suggested previously by Boinett et al. 2019 [28].

To visualize the physical alteration of the cell membrane when exposed to colistin, TEM analysis identified varying levels of membrane perforation, from partially disintegrated cells to complete lysis of the WT and Δ*stkR* mutant strains. As the cell surface hydrophobicity of the bacterial cell changes, so does the electronegativity and therefore the ability of colistin to attach and displace the cationic cations Ca^+^ and Mg^+^ causing blebbing events on the cell surface. Under sub-MIC treatment with colistin, the WT strain displayed a significant increase in membrane perturbation/protrusion known as blebbing events compared to the Δ*stkR* mutant strain (Figure 3C,D). These results are supported by other studies investigating the effect of colistin on the membrane showing envelope disruption and outer membrane collapse [23,78]. 

The increased substitution of lipid A by pEtN through transcription of *pmrC*, *eptA* a *pmrC* homologue, or the plasmid mediated *mcr-1* gene, results in increased colistin resistance [31,35,36,83]. Plasmids carrying MCR-1, a pEtN transferase enzyme that contributes to colistin resistance, have been identified in *E. coli*, *K. pneumoniae* and the *Acinetobacter* species [79]. Genome analysis did not identify this plasmid in the *A. baumannii* strain 04117201 nor the Δ*stkR* derivative (data not shown). The *pmrC* homologue *eptA* has been associated with an IS element (IS*Aba125*) insertion into a H-NS regulator gene altering transcription of *eptA*. However, no IS element was found within H-NS identified in the Δ*stkR* mutant strain, therefore we discounted these mechanisms as possible means of colistin resistance [83,84,85,86,87,88]. Similarly, an alternate means of colistin resistance is the complete loss of lipopolysaccharide/lipooligosaccharide, achieved by insertion elements affecting the transcription of the *lpxA* genes [89]. Whole genome sequencing confirmed that this mutation was not present and there were no other mutations likely to cause the phenotypes observed. 

The *pmrCAB* system was examined by qRT-PCR, as it plays a major role in conferring colistin resistance by the modification of lipid A with pEtN. The PmrAB TCS can be regulated by other two-component systems as seen in *Salmonella enterica* and *K. pneumoniae*. In these cases, the PhoPQ system leads to the activation of a small polypeptide known as *pmrD*, which binds to *pmrA* and stabilizes it in its phosphorylated state leading to an increase in transcription of *pmrC* [30]. Even though a *pmrD* homologue was not identified in *A. baumannii* 04117201, the cross-regulation of *pmrCAB* is not limited to interactions with the PhoPQ system but can be further complicated by other TCS (e.g., *preAB* in *S. enterica*) [90]. This PreAB TCS is like the *qseBC* system found in *E. coli* and appears to be able to influence the expression of *pmrCAB* as well, suggesting that the *pmrCAB* system could be regulated by several TCS.

We showed that the levels of *pmrA*, *pmrB*, and *pmrC* were significantly increased in Δ*stkR* compared to the WT when exposed to colistin. Additionally, we demonstrated that the PmrAB TCS is a likely contributor to colistin resistance and that the StkSR two-component system is a probable influence on the regulation of the PmrAB system. Taken collectively, these data could explain the modified colistin resistance seen when *stkR* is inactivated [90,91]. Lastly, an increase in colistin resistance through the PmrAB TCS is reported to lead to a decrease in fitness [30,92]. However, we have yet to identify any decrease in fitness of the Δ*stkR* mutant. 

## 5. Conclusions

This is the first study to describe the novel TCS StkSR identified in an *A. baumannii* strain isolated from an Australian hospital. We have demonstrated that the observed increase in colistin resistance seen for the Δ*stkR* derivative of this isolate is not due to the loss of lipid A but correlates to the increased transcription of the *pmrCAB* operon which is linked to the modification of lipid A. This modification decreases the overall membrane electronegativity and therefore decreases the cells affinity to the colistin compound. Colistin represents a “last resort” antibiotic used for the treatment of severe *A. baumannii* infections, and understanding the resistance mechanisms to this clinically important compound is vital for its continued application. 

## Figures and Tables

**Table 1:** Antimicrobial resistance profiles of *Acinetobacter baumannii* clinical strains.

	*A. baumannii* Strains										
Compound *^ab^*	ATCC 17978	04117201	1172312	WM98 *^c^*	08315000	04397670	9030751	1077697	9038266	08325050	083217005
KAN *^c^*	R	R	R	S	S	R	R	S	R	S	R
ERY	S	S	S	S	R	S	S	I	S	I	R
TET	S	R	R	I	R	I	R	I	R	R	R
AMP	250	>500	>500	250	>500	>500	>500	>500	>500	>500	>500
CIP	3.25	30	30	3.25	>240	60	60	60	60	>240	>240
CST	4	8	8	16	8	8	32	32	16	4	4
TEL	0.93	1.87	1.87	1.87	3.75	3.75	1.87	1.87	3.75	3.75	3.75
GEN	32	500	500	16	125	250	125	125	250	125	125
PMB	8	16	16	16	8	16	32	32	8	8	8
RIF	8	8	8	8	>64	4	4	4	4	4	4
H_2_O_2_ *^d^*	125	250	250	500	250	125	250	250	250	125	250

*^a^* KAN, kanamycin; ERY, erythromycin; TET, tetracycline; AMP, ampicillin; CIP, ciprofloxacin; CST, colistin; TEL, tellurite; GEN, gentamicin; PMB, polymyxin B; RIF, rifampicin; H_2_O_2_, hydrogen peroxide. *^b^* (µg/mL). *^c^* R, Resistant; I, Intermediate; S, Susceptible; determined by Kirby Bauer against the CLSI standards. *^d^* (nM).

**Table 2:** Response regulators identified in the clinical *Acinetobacter baumannii* 04117201.

Peg ^#^ in 04117201	Location in 04117201	A1S + ^#^ in ATCC 17978	Location in ATCC 17978	Present in SDF	Response Regulator *^a^*	Description
25	18192–18935	A1S_1753	2038890–2044193	N	AdeR	TCS, known as AdeRS, involved in antibiotic resistance and AdeABC efflux pump [58]
95	40498–39824	A1S_2751	3193938- 3189264	Y	PmrA	TCS, known as PmrAB involved in lipid A modification [59]
226	180539–179853	A1S_2883	3334601–3333044	Y	BaeR	TCS known as BaeRS involved in chemical transport and regulation+ of AdeABC and AdeIJK pumps [60]
519	97888–98604	A1S_2137	2497340–2492622	Y	KdpE	TCS, known as KdpED involved in potassium transport [61]
838	118775–122230	A1S_1394	1636507–1641464	N	StkR	TCS known as GerE (renamed here as StkSR)
1104	21883–21119	A1S_3229	3720752–3725517	Y	OmpR	TCS, EnvZ-OmpR, involved in osmotic stress [62]
1270	84347–85009	A1S_2288	2655031–2650369	Y	QseB	TCS, known as QseBC involved in biofilm formation [63]
1649	134448–135158	A1S_3375	3881425–3886134	Y	PhoB	TCS, known as PhoRB involved in phosphate stress and quorum sensing [64]
1825	90896–91486	A1S_2006	2319938–2324522	N	NasT	Orphan response regulator known as NasT; no histidine kinase identifiable up or down stream
1962	98859–99575	A1S_0748	887026–892617	Y	RstA	TCS, known as BfmR involved in biofilm formation [65]
2189	37589–36954	A1S_0233	259526–264163	N	PilR	TCS, known as PilR involved in Type 4 fimbriae expression [66]
2213	62365–63105	A1S_0261	284993–289733	Y	AlgB	TCS, known as AlgBZ involved in alginate biosynthesis [67]
2465	1290–2258	A1S_0621	670111–674210	N	RsbU	Orphan response regulator known as RsbU; no histidine kinase identifiable up or down stream [68]

*^a^* Annotated by Rapid Annotation using subsystem technology (RAST) https://rast.nmpdr.org/ (accessed on 15 March 2015). *^#^* Locus tag number.

**Table 3:** Antimicrobial resistance profile of the *Acinetobacter baumannii* 04117201 and Δ*stkR* mutant.

	*Acinetobacter baumannii* Strain		
Compound	WT (µg/mL)	Δ*stkR* (µg/mL)	Antibiotic Family Group
Novobiocin	31	31	Aminocoumaria
Amikacin	10	10	Aminoglycoside
Gentamicin	500	500	Aminoglycoside
Kanamycin	3000	>3000	Aminoglycoside
Streptomycin	>300	>300	Aminoglycoside
Chloramphenicol;	5	5–10	Amphenicol
Rifampicin	4	4–8	Ansamycins
Ampicillin	>500	>500	Beta-lactam
Ciprofloxacin	30	30	Carboxy fluoroquinoline
Chlorhexidine	7.5–15	15	Chlorobenzenes
Triclosan	0.15–0.65	0.31–0.65	Diphenyl ethers
Tellurite	1.87	1.87	Metal
Triton X100	64	64	Nonionic surfactant
Pentamidine	125	125	Phenol ether
Colistin	8	16	Polymyxin
Polymyxin	16	32	Polymyxin
Nalidixic acid	1250	1250	Quinolone

**Table 4:** Single nucleotide polymorphisms regions identified in the Δ*stkR* mutant compared to the WT strain.

SNP Position *^a^*	Codon in WT	Codon in Δ*stkR*	Amino Acid in WT	Amino Acid in Δ*stkR*	Annotation of Region *^b^*
176528	-	-	-	-	Non-coding region
538323	GAG	GGG	Glu	Gly	Hypothetical protein (Repeat region)
1001055	-	-	-	-	Non-coding region
1058011	-	-	-	-	Non-coding region
1098529	-	-	-	-	Non-coding region
1150782	TAA	CAA	Stop	Gln	Aldehyde dehydrogenase
1590889	GCT	GCC	Ala	Ala	Hypothetical protein
2730447	TTT	TTC	Phe	Phe	Hypothetical protein
2796261	-	-	-	-	Non-coding region
3132365	TTC	TCC	Phe	Ser	Phenazine biosynthesis protein (PhzF)
3201037	CCC	TCC	Pro	Ser	Repeat region
3361708	-	-	-	-	Non-coding region
3370031	-	-	-	-	Non-coding region
3445432	AAA	GAA	Lys	Glu	Alcyl-CoA Dehydrogenase

*^a^* SNP positions identified with SAMtools v1.9 and VCFools v0.11.17. *^b^* Annotation of region undertaken using Prokka.

## Supplementary Materials

The following are available online at https://www.mdpi.com/article/10.3390/microorganisms10050985/s1, Figure S1: Growth analysis of *A. baumannii* 04117201 and Δ*stkR* mutant strains, Table S1: Oligonucleotides used in this study.
