# Supplementary material for: The StkSR Two-Component System Influences Colistin Resistance in Acinetobacter baumannii
Source: Microorganisms. 2022 May 8;10(5):985. doi: 10.3390/microorganisms10050985 (PMC9146086; doi:10.3390/microorganisms10050985)
Supplement: Supplementary file 1 [file microorganisms-10-00985-s001.zip › microorganisms-1637641-supplementary.pdf]

(A)

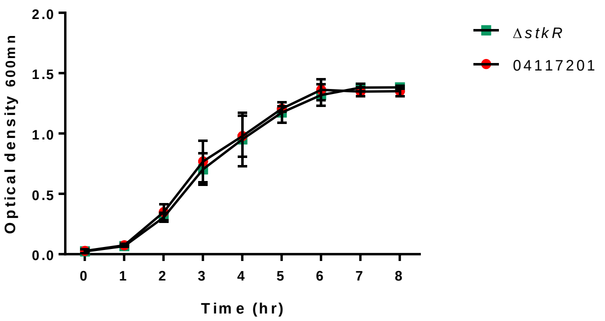

(B)

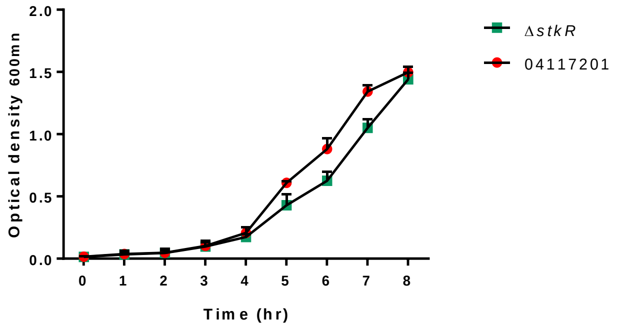

(C)

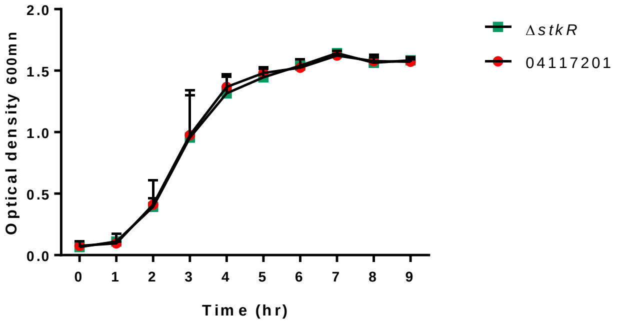

(D)

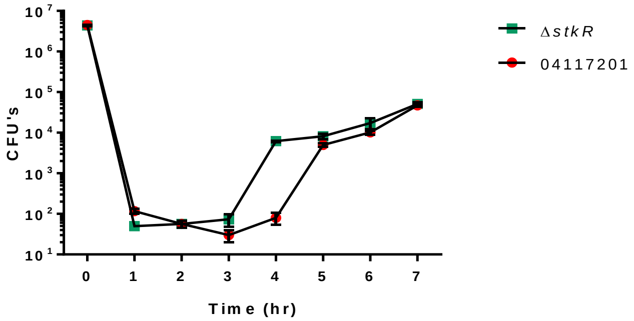

Supplemental Figure S1 Growth analysis of *A. baumannii* 04117201 and Δ*stkR* mutant strains. Samples of bacterial cells were taken every hour and growth determined spectroscopically at OD_{600} or by CFU plate count; red circles represent *A. baumannii* 04117201 and green squares the Δ*stkR* mutant strain. Cells were grown in (**A**) Mueller Hinton medium, (**B**) M9 medium, (**C**) lung medium and (**D**) whole sheep's blood. Bars represent the standard deviation of three separate experiments.

Supplemental Table S1 Oligonucleotides used in this study

**Name**	**Forward Primer**[a]	**Reverse Primer**[a]
**Cloning**		
stkR flanking UP	GAGAtctagaCCGCACTACGTTCGTACCGAC	GAGAggatccCGCCTGTTCAAGCTGTTC
stkR flanking Down	GAGAggtaccCAGACTCACTGCTAAAAATAG	GAGAgagctcGCTTCAGCCATATAGCTTC
Erythromycin resistance	GAGAggatccCTTAAGAGTGTGTTGATAGTGC	GAGAggatccAGAATTATTTCCTCCCG
**Sequencing**		
Up flanking region	GGAGATAAGTTGGGCTTGGTG	CCAATCTCTACTCCTGTTTC
Down flanking region	CTAATGCCTATGTTACTAAG	GAGGAATTCATTGTGAATATTC
pEX18Tc	CCTCTTCGCTATTACGCCAG	GTTGTGTGGAATTGTGAGCG
Erythromycin cartridge	CTTAAGAGTGTGTTGATAGTGC	AGAATTATTTCCTCCCG
stkR	ACGGTTAATTCATAAGCAATGAGT	GTGAAACTCTTGCACCAAACT
PmrA1.2	CCCATGTAAACTAAAGCGAGCC	CATCGACTTCTTGAAGTGCAACC
PmrB3.4	GGGCACCTCAATTTTCAGTGTC	GCTGAATACGCGCCAAACC
PmrB5.6	GCCGATGCTGCTCATGAATT	TTGGGCGCAAATGATGC
**qRT-PCR**		
16 S	CAGCTCGTGTCGTGAGATGT	CGTAAGGGCCATGATGACTT
*pmr*A	GAGGTGGAATGGGTCAAT	GGTCTGCTTTAAAACTTGC
*pmr*B	GGTTGCACTTCAAGAAGTC	TAAACAGATCTTCTTCGTGG
*pmr*C	CCATATTTTGGAGTAAAGGC	TTTTGGCAGTCCATTTCC

[a]Lowercase letters represent restriction endonuclease sites used for cloning the PCR amplified fragment.
